# Leukocyte Telomere Length and Long-Term Clinical Outcomes in Women with Systemic Lupus Erythematosus: A Prospective Cohort Study

**DOI:** 10.3390/jcm15124644

**Published:** 2026-06-15

**Authors:** Leyre Riancho-Zarrabeitia, Nuria Vegas-Revenga, Lucía C. Domínguez-Casas, Alfonso Corrales, Carolina Sañudo, Javier Riancho, Carmen Bejerano, Iñigo Gonzalez-Mazón, Ricardo Blanco

**Affiliations:** 1 Rheumatology Department, Hospital General Sierrallana, 39300 Torrelavega, Spain; 2 IDIVAL, 39011 Santander, Spain; 3 Department of Medicine and Psychiatry, Universidad de Cantabria, 39005 Santander, Spain; 4 Division of Rheumatology, Galdakao Usansolo University Hospital, Euskal Herriko Unibertsitatea, Biobizkaia Health Research Institute, 48960 Galdakao, Spain; 5 Rheumatology Department, Hospital San Agustin, 33400 Avilés, Spain; 6 Rheumatology Department, University Hospital Marqués de Valdecilla, 39008 Santander, Spain; 7 Neurology Department, Hospital General Sierrallana, 39300 Torrelavega, Spain; 8 CIBERNED, 28029 Madrid, Spain

**Keywords:** systemic lupus erythematosus, leukocyte telomere length, immunosenescence, cardiovascular risk, chronic kidney disease, biomarkers, cohort study

## Abstract

**Background/Objectives**: Leukocyte telomere length (TL) is a marker of biological aging associated with cardiovascular disease, chronic kidney disease, and malignancy in the general population. Its long-term prognostic significance in systemic lupus erythematosus (SLE) remains unclear. We aimed to evaluate the association between baseline TL and long-term clinical outcomes in patients with SLE. **Methods**: Prospective cohort study including 97 Caucasian women with SLE. Relative TL was measured in whole blood using quantitative polymerase chain reaction (qPCR) at baseline. A control group of 50 healthy Caucasian women from the same geographical region was included for comparison. Patients were followed for a mean of 9.7 ± 2.8 years. Outcomes included thrombotic cardiovascular events, damage accrual, incident malignancy, and chronic kidney disease. Associations were assessed using multivariable regression models adjusted for potential confounders. **Results**: Mean age was 51.6 ± 13.8 years and mean relative TL was 4.3 ± 1.0. Relative TL was inversely associated with age (β = −0.20, *p* = 0.048) and was shorter in patients with hematological manifestations (*p* = 0.038). No differences in relative TL were observed between SLE patients and controls. Relative TL was not associated with disease activity, cumulative damage, cardiovascular risk factors, vitamin D levels, or subclinical atherosclerosis. During follow-up, 13.4% of patients experienced cardiovascular events, 10.3% developed malignancy, and 11.3% developed chronic kidney disease. Relative TL was initially associated with long-term damage accrual, glomerular filtration rate and cardiovascular events; however, after adjustment for age, only the association with glomerular filtration rate remained at the limit of statistical significance (*p* = 0.05). **Conclusions**: In this prospective cohort, relative TL was primarily associated with aging, hematological manifestations, and glomerular filtration rate, but not with disease activity or most long-term clinical outcomes. These findings suggest that TL reflects biological aging rather than disease-specific processes and has limited utility as a prognostic biomarker in SLE.

## 1. Introduction

Systemic lupus erythematosus (SLE) is a chronic autoimmune disease characterized by immune dysregulation, persistent inflammation, and multisystem involvement. Despite improved survival, patients with SLE remain at increased risk of long-term complications, including accelerated cardiovascular disease [1], chronic kidney disease (CKD) [2], and malignancy [3]. These outcomes reflect the complex interplay between immune activation, oxidative stress, metabolic factors, and biological aging.

Telomeres are repetitive nucleotide sequences located at the ends of chromosomes that preserve genomic integrity and regulate cellular replicative capacity. Progressive telomere shortening occurs with cell division and is accelerated by oxidative stress and chronic inflammation. Critically short telomeres induce cellular senescence and genomic instability, linking telomere length (TL) to biological aging and age-related disease.

The relationship between TL and SLE is complex. Mendelian randomization studies suggest that genetically determined longer telomeres are associated with an increased risk of developing SLE [4,5,6]. In contrast, observational studies show that patients with established SLE have shorter telomeres compared with healthy controls [7,8,9,10,11]. Several studies have inconsistently linked telomere shortening to different clinical and immunological features of SLE such as reduced muscle strength [12], anxiety stress [13], anti-Ro antibodies [14], and vitamin D deficiency [15].

In the general population, leukocyte TL is considered a biomarker of biological aging and has been robustly associated with cardiovascular disease [16,17]. However, results in subclinical atherosclerosis are heterogeneous [18,19,20]. Similarly, multiple studies have demonstrated an association between shorter TL and chronic kidney disease [21], the degree of renal decline [22], and mortality [23]. The relationship between TL and cancer is bidirectional. Short telomeres promote chromosomal instability and have been associated with increased cancer incidence and mortality [24,25]. Conversely, genetically determined longer TL increases the risk of specific malignancies, including thyroid and pancreatic cancer, melanoma, and lung adenocarcinoma [26,27]. These findings highlight the complex role of telomere biology in carcinogenesis.

Despite this extensive literature in general population, the long-term prognostic significance of TL in SLE remains unclear. Most previous studies have been cross-sectional and have not evaluated hard clinical outcomes over extended follow-up. Whether TL predicts major cardiovascular events, cumulative organ damage, incident malignancy, or chronic kidney disease in SLE has not been adequately investigated in prospective cohorts.

The present study aimed to assess the association between baseline leukocyte TL and 10-year outcomes in a well-characterized cohort of women with SLE. Specifically, we examined whether TL predicts: (1) cardiovascular events, (2) disease-related damage accrual, (3) incident neoplasms, and (4) chronic kidney disease during long-term follow-up.

## 2. Patients and Methods

### 2.1. Study Design and Population

This prospective cohort study included 97 consecutive female SLE patients fulfilling the ACR 1997 classification criteria [17] for SLE who attended the University Hospital Marqués de Valdecilla between June 2014–July 2015. Formal retrospective application of the 2019 EULAR/ACR classification criteria was not possible because some of the required variables were not systematically collected. Inclusion criteria were: (1) diagnosis of SLE, (2) female sex, (3) age ≥ 18 years, (4) Caucasian origin, and (5) provision of written informed consent. All participants provided written informed consent. The study was approved by the Ethics Committee of Cantabria (protocol code 2014.029) and was conducted in accordance with the Declaration of Helsinki and the General Data Protection Regulation (GDPR). Patient confidentiality was maintained throughout the study.

The SLE cohort was compared with controls selected from volunteers in the general population participating in a bone health study. The control group consisted of 50 healthy Caucasian women from the same geographical region as the patients, with a mean age of 56 ± 19 years, which was not significantly different from that of the SLE cohort (*p* = 0.19). Individuals with known autoimmune diseases were excluded from the control cohort.

### 2.2. Clinical and Laboratory Assessment

Baseline data included demographics, disease duration, and cardiovascular risk factors such as smoking, hypertension, diabetes, dyslipidemia, and metabolic syndrome. Subclinical atherosclerosis was assessed using carotid ultrasound to evaluate intima–media thickness (IMT) and plaque presence. Disease activity and damage were measured using SLEDAI and SLICC/ACR-DI indices.

Primary outcomes were: (1) thrombotic events, including arterial events (coronary artery disease, stroke, peripheral arterial thrombosis, peripheral artery disease) and venous thrombosis (deep vein thrombosis, pulmonary embolism, other venous events), (2) damage accrual according to SLICC/ACR-DI, (3) neoplasia, and (4) chronic kidney disease. Events were confirmed by medical record review and imaging studies.

### 2.3. Telomere Length

Leukocyte TL was measured in genomic DNA extracted from peripheral whole blood samples obtained at baseline using the Blood GenomicPrep Mini Spin Kit (Cytiva Marlborough, MA, USA). DNA concentration was quantified with a Qubit fluorometer (Invitrogen, Waltham, MA, USA), and samples were normalized to 60 ng per reaction. Relative TL was assessed by quantitative PCR according to a previously published method [28], with minor modifications. Telomere and albumin (single-copy reference gene) amplifications were performed in separate reactions using Power SYBR™ Green Master Mix on a QuantStudio™ 5 Real-Time PCR System (Applied Biosystems, Waltham, MA, USA). All samples were analyzed in triplicate and independently repeated in three different plates to ensure reproducibility. A pooled DNA sample was included in each run as an internal control for inter-run consistency. Relative TL was calculated from the difference between telomere and albumin threshold cycles.

### 2.4. Statistical Analysis

Patients were followed longitudinally, and electronic health records were reviewed. Follow-up ended on 1 January 2026.

Continuous variables are presented as mean ± standard deviation (SD) or median and interquartile range. Associations between TL, as a continuous variable, and other continuous variables were assessed by correlation and regression analysis. The associations between categorical variables were compared using the χ^2^ test or Fisher’s exact test, as appropriate. Odds ratios (ORs) and the 95% confidence intervals (CIs) were estimated using logistic regression models, either unadjusted or adjusted for potential confounders. The main outcome variables were cardiovascular events, neoplasia and death. The main predictor was telomere length (in quartiles). For the multivariate analyses, we included in the models the variables with a *p*-value < 0.20 in the univariate analyses and used both backward and forward selection strategies to confirm the consistency of associations. Finally, we estimated the ORs adjusted for covariables with *p*-values < 0.05.

## 3. Results

A total of 97 women with SLE were included in the study. The mean age at baseline was 52 ± 14 years, and the mean disease duration was 10.0 (4.3–15.0) years. Baseline characteristics of population are summarized in Table 1.

The mean follow-up duration was 9.7 ± 2.8 years (range 0.6–11.5 years). Seven patients were lost to follow-up after a mean duration of 4.0 ± 4.4 years. Among those (n = 90) completing follow-up, the mean duration was 10.2 ± 2.1 years. Overall, 13 patients (13.4%) experienced at least one thrombotic cardiovascular event during the follow-up. A total of 10 patients (10.3%) developed a neoplasia during follow-up and nine patients died (9.3%). The most common causes of death were cardiovascular events (3 patients), cancer (2), heart failure (2), sepsis (1), and upper digestive bleeding (1). The mean estimated glomerular filtration rate (eGFR) was 84 ± 18 mL/min at the end of follow-up, and 11 (11.3%) had chronic kidney disease (as eGFR < 60 mL/min1.73 m^2^). Regarding disease damage the mean SLICC/ACR Damage Index score increased from 1.1 ± 1.3 at baseline to 1.5 ± 1.5 at the end of follow-up.

### Telomere Length

**Figure 1:**
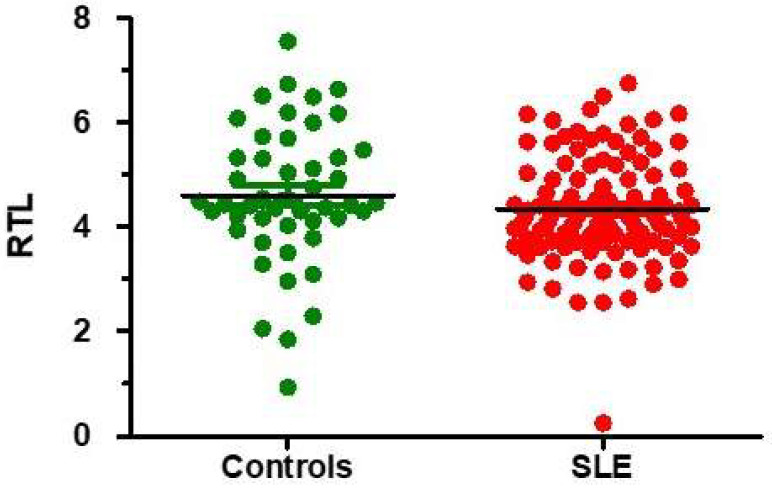
Relative leukocyte telomere length in patients with SLE and controls. Horizontal bars indicate the mean RTL value for each group.

There were no significant differences in TL between SLE patients and healthy controls (4.3 ± 1.0 vs. 4.6 ± 1.3, *p* = 0.15) (Figure 1).

**Figure 2:**
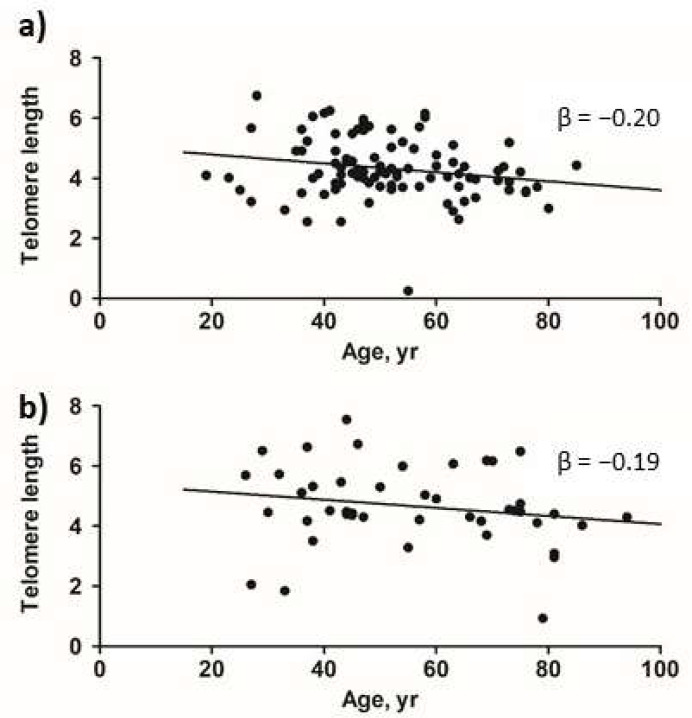
Relationship between leukocyte telomere length and age in (**a**) SLE patients and (**b**) controls. TL was inversely associated with age in SLE (β = −0.20, *p* = 0.048) and controls (β = −0.19, *p* = 0.22).

TL was inversely associated with age in SLE patients (β = −0.20, *p* = 0.048), as well as in controls (Figure 2).

**Figure 3:**
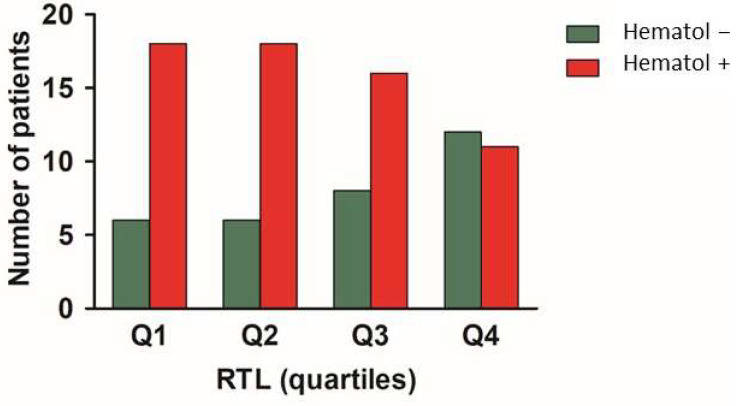
Distribution of patients with and without hematological manifestations according to relative telomere length quartiles.

When we compared TL in different subgroups of patients with SLE, we found statistically significant differences between patients with and without hematological manifestations (*p* = 0.038). There was a significant association of relative TL quartiles and the prevalence of hematological manifestations (*p* for trend = 0.042). Thus, compared with patients in quartile 4 RTL (longest telomeres), those in quartile 3 had a 2.2 OR, while those in quartiles 2 and 1 had a 3.3 OR for hematological manifestations (Figure 3).

TL was not associated with cardiovascular risk factors, other disease-specific manifestations, disease activity (SLEDAI), cumulative damage (SLICC/ACR DI), or disease duration (all *p* > 0.05). No significant associations were observed between TL and vitamin D levels or other laboratory parameters including adipokines, endothelial activation markers, or vascular biomarkers. TL was not associated with the presence of carotid plaque or IMT at baseline.

The long-term follow-up of patients allowed us to explore the association of TL with different variables of patient status Table 2. We found an association of TL with the final SLICC/ACR DI score (*p* = 0.029), the eGFR (*p* = 0.035), and cardiovascular events during follow-up (*p* = 0.058). However, only the association between TL and eGFR remained in the limit of statistical significance after including age as a covariate. No association was found between TL and the development of new carotid plaque or malignancy.

## 4. Discussion

In this prospective cohort of women with SLE we found that leukocyte TL measured in whole blood was primarily associated with chronological age and with hematological manifestations. In contrast, TL was not associated with disease activity, cumulative damage, vitamin D levels, or subclinical atherosclerosis. Regarding long-term outcomes TL was associated with renal function while no association was found with disease damage, incident cardiovascular events, or malignancy. Importantly, TL did not differ between SLE and controls in our cohort, and TL showed a similar age-dependent decline in both populations.

The absence of significant differences in TL between SLE and controls should be interpreted in the context of previous studies suggesting that telomere shortening in SLE may be age-dependent. Several investigations have reported that differences in TL appear in younger individuals [7,8,11], whereas they tend to diminish or disappear with increasing chronological age. This phenomenon has been attributed to the progressive telomere attrition that occurs in healthy individuals over time, leading to a convergence of TL between patients and controls at older ages. However, in our cohort, we specifically evaluated this hypothesis and found no significant differences in TL between SLE patients and controls even when restricting the analysis to younger individuals, in accordance with some previous authors [29]. In addition, variability across studies may be partly explained by differences in ethnic background. While telomere shortening has been reported across multiple populations [9], its magnitude appears to be greater in certain ethnic groups, particularly in African American patients, who often exhibit more severe disease phenotypes [10,15]. In contrast, studies conducted in predominantly Caucasian populations [29] have sometimes failed to detect significant differences between patients and controls. Furthermore, methodological aspects, including the use of whole blood versus specific immune cell subsets, may influence the detection of telomere alterations. Taken together, these observations highlight the heterogeneity of telomere dynamics in SLE and support the interpretation that the lack of differences observed in our study reflects true biological variability rather than insufficient analytical sensitivity.

The association between shorter TL and biological age is well known in general population and indeed TL is a marker of biological age [30]. Interestingly some authors found a weak [8] or no correlation in SLE patients [7,14], probably influenced by the effect of disease in TL independently of chronological age. Moreover, the association of TL with eGFR is noteworthy and consistent with evidence from the general population linking telomere shortening with chronic kidney disease and renal decline [21,23]. Mendelian randomization studies [21] have demonstrated that telomere attrition not only contributes to the development of chronic kidney disease but that declining renal function itself may further accelerate telomere shortening. This may reflect shared mechanisms such as oxidative stress, inflammation, and replicative senescence in renal compartments. In contrast, the association between lupus nephritis history or proteinuria and TL has not been found [8,14]. This discrepancy may suggest that TL is more sensitive to systemic cellular turnover than to organ-specific inflammatory manifestation.

A key finding of our study is the association between TL and hematological manifestations. Hematological involvement is characterized by cytopenias due to autoantibodies or drugs effects that cause peripheral destruction and compensatory hematopoietic turnover, directly affecting circulating leukocytes, which may lead to accelerated telomere attrition. This supports the concept that telomere shortening reflects cumulative hematopoietic stress and immune cell turnover.

However, we did not find an association between TL and SLE activity or cumulative damage. This is consistent with several prior studies [7,11,12,13,14] that did not find an association between SLEDAI or SLICC/ACR-DI. Collectively, these findings suggest that TL does not reflect the current inflammatory state. Although cumulative damage is commonly interpreted as a marker of long-term disease burden, it may not adequately reflect the complex biological pathways underlying telomere attrition. Telomere shortening is driven by a combination of oxidative stress, replicative senescence, immune dysregulation, and genetic factors, which are not fully captured by clinical indices of damage.

Despite robust evidence linking leukocyte TL to cardiovascular disease in the general population [16,17], its association with subclinical atherosclerosis remains inconsistent [18,31]. In our study, we observed a non-significant trend between shorter TL and the risk of subsequent cardiovascular events; however, no association was found between TL and markers of subclinical atherosclerosis, including carotid plaque and carotid intima–media thickness. Data specifically addressing this relationship in patients with SLE are extremely limited. Only Skamra et al. [11] similarly reported that TL was not independently associated with carotid plaque after adjustment for age.

Telomere biology has a complex and often paradoxical relationship with cancer, as both excessively short and relatively long telomeres have been implicated in tumor development [25,26,32,33]. This suggests a potential non-linear or U-shaped relationship between TL and cancer risk, depending on tumor type and biological context. In our cohort, TL was not associated with incident malignancy during follow-up. However, the number of incidental neoplasia was low, which may have limited the ability to detect modest associations.

We did not observe any association between leukocyte TL and vitamin D levels in our cohort. In the general population, serum 25-hydroxyvitamin D has been positively associated with TL, particularly in women, adults, and individuals with vitamin D deficiency, whereas this relationship was not found in men, children, and those with sufficient vitamin D levels [34]. In SLE patients, however, available evidence is heterogeneous, with prior studies reporting inconsistent or population-specific associations [11,15].

The strengths of our study include its prospective design, long-term follow-up, well-characterized cohort, and comprehensive assessment of clinically relevant outcomes. Unlike most previous studies, which were cross-sectional, our study directly evaluated the prognostic value of TL. Several limitations should be acknowledged. First, TL was measured at a single time point, precluding assessment of telomere attrition rates over time. Second, the use of whole blood may have limited sensitivity to detect cell-specific effects. Third, the cohort consisted exclusively of Caucasian women, which may limit generalizability. In addition, although the cohort was followed prospectively for nearly 10 years, the absolute number of several clinically relevant outcomes, including malignancies, deaths, thrombotic events, and incident chronic kidney disease, was relatively low. As a result, the statistical power to detect modest or moderate associations between TL and these outcomes may have been limited. This increases the risk of type II error, whereby true associations may not have been identified because of insufficient event numbers. Consequently, the lack of statistically significant associations for certain outcomes should be interpreted cautiously and should not be regarded as definitive evidence that no biological or clinically meaningful relationship exists between TL and these events. Furthermore, the limited number of outcome events may have reduced the precision of risk estimates, potentially leading to wider confidence intervals and greater uncertainty regarding the magnitude of any underlying associations. Additional studies with larger sample sizes and a greater number of clinical events will be necessary to more robustly evaluate the prognostic relevance of TL for these less frequent outcomes. Finally, the observational nature of the study precludes any inference of causality. Although multivariable adjustment was performed to account for potential confounders, residual confounding arising from unmeasured or incompletely measured variables cannot be entirely excluded. Therefore, the observed associations should be interpreted as exploratory and hypothesis-generating rather than definitive. In particular, further validation in independent cohorts is required before TL can be considered a reliable prognostic biomarker for long-term clinical outcomes in patients with SLE.

## 5. Conclusions

In conclusion, leukocyte TL was primarily associated with chronological age and hematological manifestations, but not with disease activity, cumulative damage, or long-term outcomes except for GFR. These findings suggest that TL reflects systemic biological aging rather than disease-specific processes in SLE. However, given the relatively small number of outcome events, these results should be considered exploratory, and the prognostic value of TL for long-term clinical outcomes in SLE requires further confirmation.

## Figures and Tables

**Table 1:** Baseline characteristics of SLE patients.

	Total (*n* = 97)
Age, mean ± SD (years)	51.6 ± 13.8
Disease duration, median (RIC) (years)	10.0 (4.3–15.0)
**Cardiovascular risk factors**	
Current smoker/Previous smoker, (n, %)	23 (24)/24 (25)
Hypertension, n (%)	27 (28)
Diabetes mellitus, n (%)	3 (3)
Dyslipidemia, n (%)	20 (21)
Metabolic syndrome, n (%)	17 (18)
Obesity, n (%)	21 (22)
Abdominal perimeter (cm), mean ± SD	90 ± 13
Previous CV events, n (%)	7 (7)
**Subclinical atherosclerosis**	
Carotid plaque, n (%)	43 (44)
IMT (µm), mean ± SD	663 ± 140
**SLE manifestations, n (%)**	
Articular	89 (91.5)
Cutaneous	94 (97)
Hematologic	64 (66)
Renal	14 (14.6)
Neuropsychiatric	8 (8.3)
Antiphospholipid antibodies, n (%)	42 (43)
SLEDAI, median (RIC)	2 (0–4)
SLICC/ACR-DI, median (RIC)	1 (0–2)
Vitamin D (ng/mL), median (RIC)	19.6 (13.1–25.6)
Telomere length, mean ± SD, arbitrary units	4.3 ± 1.0

SD: standard deviation, CV: cardiovascular, IMT: intima–media thickness, SLE: systemic lupus erythematosus, SLEDAI: systemic lupus erythematosus disease activity index, SLICC/ACR-DI: systemic Lupus International Collaborating Clinics/American College of RheumatologyDamage Index.

**Table 2:** Association between telomere length and long-term clinical outcomes: crude and age-adjusted analyses.

**Variable**	**r**	***p***	**Adjusted r**	***p***
eGFR	0.23	0.035	0.21	0.05
SLICC/ACR-DI	−0.24	0.029	−0.17	0.12
	**OR**	***p***	**Adjusted OR**	***p***
Cardiovascular events	0.49 (0.24–1.02)	0.058	0.61 (0.27–1.41)	0.25
Malignancy	0.93 (0.51–1.67)	0.80	0.93 (0.51–1.70)	0.82
Mortality	0.71 (0.37–1.36)	0.30	0.93 (0.45–1.94)	0.86

## Data Availability

Data are available upon reasonable request.

## Abbreviations

The following abbreviations are used in this manuscript:

ACR	American College of Rheumatology
CKD	Chronic Kidney Disease
CV	Cardiovascular
eGFR	Estimated Glomerular Filtration Rate
IMT	Intima–Media Thickness
OR	Odds Ratio
SD	Standard Deviation
SLE	Systemic Lupus Erythematosus
SLEDAI	Systemic Lupus Erythematosus Disease Activity Index
SLICC/ACR-DI	Systemic Lupus International Collaborating Clinics/American College of Rheumatology Damage Index
TL	Telomere Length
